# Effect of Nitrogen, Salinity, and Light Intensity on the Biomass Composition of *Nephroselmis* sp.: Optimization of Lipids Accumulation (Including EPA)

**DOI:** 10.3390/md21060331

**Published:** 2023-05-28

**Authors:** Savvas Giannis Mastropetros, Konstantina Tsigkou, Yannis Cladas, Arun Kumar Priya, Michael Kornaros

**Affiliations:** 1 Laboratory of Biochemical Engineering & Environmental Technology (LBEET), Department of Chemical Engineering, University of Patras, 26504 Patras, Greece; savvasgiannismas@gmail.com (S.G.M.); ktsigkou@chemeng.upatras.gr (K.T.); 2 Department of Animal Production, Fisheries & Aquaculture, University of Patras, Mesolonghi, 30200 Nea Ktiria, Greece; jkladas@upatras.gr; 3 Department of Chemical Engineering, KPR Institute of Engineering and Technology, Coimbatore 641407, India; a.k.priya@kpriet.ac.in; 4 Project Prioritization, Monitoring & Evaluation and Knowledge Management Unit, ICAR-Indian Institute of Soil & Water Conservation (ICAR-IISWC), Dehradun 248195, India

**Keywords:** *Nephroselmis* sp., microalgae, biomass composition, lipids accumulation, dietary supplement, eicosapentaenoic acid (EPA), Box–Behnken experimental design

## Abstract

Microalgal biomass is characterized by high protein, carbohydrates, and lipids concentrations. However, their qualitative and quantitative compositions depend not only on the cultivated species but also on the cultivation conditions. Focusing on the microalgae’s ability to accumulate significant fatty acids (FAs) amounts, they can be valorized either as dietary supplements or for biofuel production, depending on the accumulated biomolecules. In this study, a local isolate (*Nephroselmis* sp.) was precultured under autotrophic conditions, while the Box–Behnken experimental design followed using the parameters of nitrogen (0–250 mg/L), salinity (30–70 ppt) and illuminance (40–260 μmol m^−2^ s^−1^) to evaluate the accumulated biomolecules, with an emphasis on the amount of FAs and its profile. Regardless of the cultivation conditions, the FAs of C14:0, C16:0, and C18:0 were found in all samples (up to 8% *w/w* in total), while the unsaturated C16:1 and C18:1 were also characterized by their high accumulations. Additionally, the polyunsaturated FAs, including the valuable C20:5n3 (EPA), had accumulated when the nitrogen concentration was sufficient, and the salinity levels remained low (30 ppt). Specifically, EPA approached 30% of the total FAs. Therefore, *Nephroselmis* sp. could be considered as an alternative EPA source compared to the already-known species used in food supplementation.

## 1. Introduction

The majority of environments and habitats worldwide, such as seawater, brackish or freshwater, rocks, land, and soil, among others, are appropriate for microalgae growth. According to the literature, they may grow when attached to substrates, form mats, or float [1]. Microalgae have exhibited a wide range of cultivation and growth conditions (temperature, nutrient availability, pH level, light intensity, and CO_2_ presence) and morphology and size. For example, species such as *Dunaliella salina* are able to tolerate and adapt to high salinity levels at a significantly wide temperature range of 0–35 °C [1,2]. It is known that, aside from their critical role in the cycles of carbon and nitrogen on Earth, they are also produced and used commercially, either in the food and pharmaceutical industries or as biofuels [3,4]. Due to the high diversity of microalgae and their various growth requirements, the isolation and selection of local strains seem to be an effective alternative for their utilization, especially in the case of outdoor cultivation. As the climate of a location can significantly impinge on the microalgal population and growth dynamics, their screening and growth investigation could enhance their positive impact on high-added value compound accumulation [5,6].

The Mediterranean region is known for exhibiting ideal climate conditions for microalgae growth, especially in the south due to the warm temperatures, which usually do not fall below 15 °C [7]. According to the literature, several microalgal and cyanobacterial species have been isolated in Greek environments and habitats by various research groups [6,8,9,10,11], usually targeting and taking advantage of the sturdy local isolates to enhance value-added compound production [6]. More specifically, 15 *Dunaliella* strains were isolated by Lortou et al. [9] from Greek saltworks, resulting in nine different species, which were *D. minutissima*, *D. parva*, *D. asymmetrica*, *D. minuta*, *D. terricola*, *D. viridis*, *D. granulata*, *D. bioculata*, and *D. polymorpha*. On the other hand, Prasinophyceae strains were isolated by Tzovenis et al. [11] from a Greek coastal lagoon. Both the *Tetraselmis* and *Pyramimonas* local isolates were presented as good alternatives for polyunsaturated FAs (PUFAs) production. Additionally, according to Gkelis et al. [10], 29 strains were isolated from freshwaters in Greece and were classified as Chroococcales, Synechococcales, and Nostocales, and reported the following strains, such as *Nostoc oryzae*, *Chlorogloeopsis fritschii,* and *Synechococcus* cf. *nidulans*. Finally, according to Hotos et al. [6], one dinoflagellate (*Amphidinium carterae*), three cyanobacteria (*Phormidium* sp., *Anabaena* sp., and *Cyanothece* sp.), and five Chlorophyta species (*Asteromonas gracilis*, *Dunaliella* sp., *Tetraselmis marina*, *Tetraselmis* sp., and *Nephroselmis* sp.) were collected from Western Greece lagoons, while their identification, morphological observation, and characterization followed.

Especially for the genus *Nephroselmis*, it is worth mentioning that it was established by Stein in 1878 on the basis of freshwater species and was named *Nephroselmis olivacea* Stein. Over time, more than 10 *Nephroselmis* species have been identified, originating either from freshwater or marine environments [6,12,13]. The general characteristics of the specific genus are as follows: (a) a flattened cell body with two flagella of different sizes (one short one at the front and one longer one at the back) that move in different directions; (b) scales on the surface, the second of which is rod-shaped; (c) two to four layers of scales on the cell surface, the second of which has an unclear shape; and (d) flagellar with a root system, which includes a rhizoplast and three microtubular roots [14]. A fairly wide range of *Nephroselmis* sp. cultivation conditions and carbon sources have been tested, targeting the enhancement of biomass production and added-value compound accumulation, mainly FAs and pigments [8,15,16,17].

Focusing on lipids accumulation, their classification refers to polar (phospholipids and glycolipids) and neutral (acylglycerols and free FAs) lipids. Their content, described as the dry weight of biomass, may vary between 1.5 and 75%, depending on the species, culture medium, and cultivation conditions. Moreso, FAs are carboxylic acids with chains of 4 to 36 C atoms and various levels of saturation (mono-, di- or even poly-saturated) [18]. Their production can be strongly influenced and manipulated due to specific (stress) condition applications. Specifically, PUFAs, mainly C20:5n3 (EPA) and C22:6n3 (DHA), have been widely recognized as essential compounds for both nutrition and health due to their participation in the metabolic pathways responsible for resolving inflammation. Furthermore, EPA is essential for several biological regulation functions, including cardiovascular disease, arrhythmia, atherosclerosis, and cancer. EPA is a PUFA that is heavily involved in the food and nutritional supplement industry, and thus its isolation from autotrophic microorganisms, such as these of the *Nephroselmis* genus, could minimize the production costs in combination with a sustainable downstream process [19,20]. Due to their importance in human wellness and health, there is high market interest worldwide, indicating that the omega-3 market size in 2019 was estimated at USD 2.49 billion, with the potential for further expansion [19].

Regarding biomass production enhancement and added-value compound accumulation, it is widely known that different cultivation strategies should be used. For instance, it was mentioned in the literature that a two-fold biomass productivity increase led to an overall biodiesel decrease of around 40%. Such an example significantly indicates the need for cultivation conditions manipulation [21,22]. More specifically, optimal conditions should be provided if the main target is biomass accumulation, while alterations to the cultivation conditions should be applied, for instance, nutrient depletion, cultivation systems, or growth modes, if the study focuses on valuable compound accumulation. For this reason, two-stage cultivation systems have been proposed in order to separate the two aforementioned phases and effectively exploit the microalgal biomass [21,23] compared to the one-stage systems. While pointing out two-stage cultivation strategies, several types have been proposed in the literature regarding inducer additions, nutrient starvation, metabolic switch, irradiation, or multi-stress applications [21].

The aim of the current study is the evaluation of a local isolate of the genus *Nephroselmis* regarding its adaptation to a range of salinity values, light intensity, and concentration of available nitrogen. This green alga was cultivated under different conditions, as they resulted from an experimental design using the Box–Behnken methodology. The biomass of *Nephroselmis* sp. gathered research interest due to the variety of the FAs content and, specifically, the existence of the nutritionally valuable EPA. It was collected after five days, and the production of FAs (including EPA), as well as proteins, carbohydrates, and total pigments, was investigated. From the above, a primary conclusion can be drawn about whether the strain under study warrants being used to recover healthy fats and whether its protein and carbohydrate contents make it intrinsically valuable as a nutritional supplement.

## 2. Results and Discussion

### 2.1. Biomass Adaptation and Lipids Concentration

To determine the concentration of each cellular component in the culture medium (proteins, carbohydrates, FAs, pigments), it was first necessary to measure the concentration of the produced biomass and analyze its composition on a dry weight basis. Table 1 presents the biomass concentration values of all the experimental scenarios resulting from the experimental design. The biomass concentration values were not statistically different after the analysis of variance and *t*-test comparison, with a significance level of 0.05. Since the substrates were inoculated by a parent autotrophic culture aiming to contain an initial biomass concentration of 100 mg L^−1^, it was observed that *Nephroselmis* sp. did not show new growth, even in the cases of nitrogen and light sufficiency. It was inferred that the local isolate used the five days spent in the new flasks as a period of adaptation to the new conditions studied. The microorganisms of the genus *Nephroselmis* have been reported regarding their lag phase ranging from 2 to 10 days, depending on the growth conditions [24]. The particular local species was also examined by Hotos et al., who observed a similar behavior during its autotrophic growth, as it took four to five days to begin the exponential phase of growth [8]. The concentration values found below 100 mg L^−1^ can be attributed both to the experimental error and the lysis of some cells, especially in cultures without a nitrogen source and/or under inadequate light. It is worth mentioning that the growth rate of microalgae is limited under nutrient and light stress; however, with the appropriate carbon-to-nitrogen ratio in the substrate, valuable biomolecules, such as FAs, can accumulate. These FAs are no longer a component of the cellular membranes but form triglycerides [25]. 

**Figure 1:**
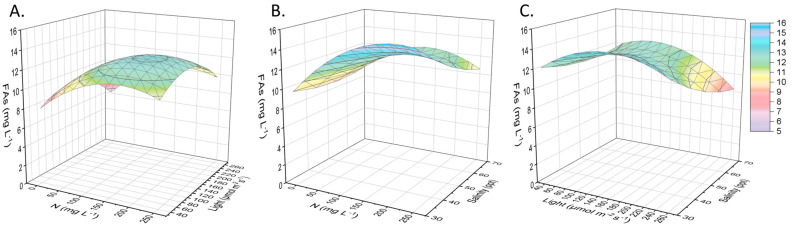
Presentation of the surface plots regarding the FAs production as a function of the initial nitrogen concentration, light intensity, and salinity. Fatty acids (mg L^−1^) related to (**A**) nitrogen concentration (mg L^−1^) and light intensity (μmol of photons m^−2^ s^−1^), given salinity of 50 ppt. (**B**) Nitrogen concentration (mg L^−1^) and salinity (ppt), given light intensity of 110 μmol of photons m^−2^ s^−1^. (**C**) Salinity (ppt) and light intensity (μmol of photons m^−2^ s^−1^), given nitrogen concentration of 125 mg L^−1^.

The FAs reached a maximum concentration of 15 mg L^−1^ (Figure 1), while their production under the tested ranges of nitrogen concentration, salinity, and light intensity can be predicted by the equations in Table 2. In the same table, it can be seen that only the parameter of nitrogen played a significant role statistically. The most encouraging results, which were related to FAs accumulation, were found in cultures that had a nitrogen content between 210 and 220 mg L^−1^ and light intensity of 150 to 170 μmol m^−2^ s^−1^ when the salinity of the substrate was adjusted to 30 ppt. Nitrogen deficiency did not favor the FAs’ synthesis as expected, and therefore, it can be assumed that *Nephroselmis* sp. consumed the intracellular lipids (initially around 20% of the dry biomass [6]) for energy during its adaptation period to the second cultivation stage. Microalgae store organic carbon for their long-term energy needs in the form of FAs. For instance, palmitic acid can be oxidized in the mitochondria to produce energy according to the reaction described by Sorgüven et al. [26]:

Palmitic acid + ATP + CoA → Palmitoyl-S-CoA + AMP + Ppi

Palmitoyl-S-CoA + 23 O_2_ + 131 Pi + 131 ADP → CoA + 131 ATP + 16CO_2_ + 146 H_2_O

In another case, when *Chlorella protothecoides* was studied, a decrease in the fatty acid content was also observed during the adaptation period from the first to the second stage of cultivation. This particular species spent 36 days in the lag phase in the substrate with limited nitrate concentration. As a result, FAs fell from 20% to below 15% of the dry biomass weight during that time [27]. *Nephroselmis* sp. has exhibited similar behavior in conditions of nitrogen deficiency but in terms of cellular pigment (chlorophylls and carotenoids) reduction [16]. Given the fixed nitrogen concentration value (Figure 1), a preference for low salinity levels was observed, and a region of optimal light intensity appeared around 150 μmol m^−2^ s^−1^ (for lipids production). It has been previously reported that the biomass lipids of *Nephroselmis* genus microalgae also reached 18.8% w/w when grown under 120 μmol of photons m^−2^ s^−1^ [15].

**Figure 2:**
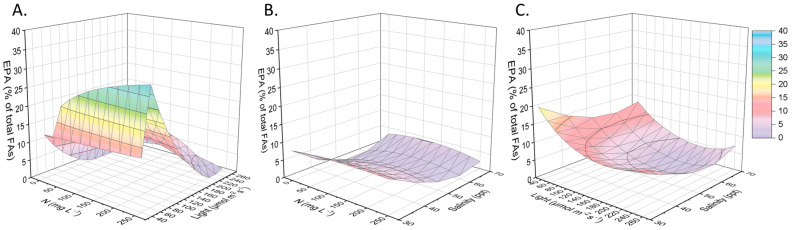
Presentation of the surface plots regarding the EPA production as a function of the initial nitrogen concentration, light intensity, and salinity. EPA (% of total FAs) related to (**A**) nitrogen concentration (mg L^−1^) and light intensity (μmol of photons m^−2^ s^−1^), given salinity of 50 ppt. (**B**) Nitrogen concentration (mg L^−1^) and salinity (ppt), given light intensity of 110 μmol of photons m^−2^ s^−1^. (**C**) Salinity (ppt) and light intensity (μmol of photons m^−2^ s^−1^), given nitrogen concentration of 125 mg L^−1^.

Furthermore, similar to the total FAs, EPA also appeared to accumulate in low salinity levels and light irradiation. It almost reached 30% of the total FAs when the cultures had light equal to 60 μmol m^−2^ s^−1^ and when the initial nitrogen concentration exceeded 100 mg L^−1^ (Figure 2). Microalgae are able to accumulate n−3 PUFAs up to 10–20% of their dry biomass, accompanied by low proliferation rates due to the nutrients’ limitations [28]. Some photosynthetic microorganisms produce EPA or docosahexaenoic acid (DHA), while others contain both valuable FAs. Regarding the EPA, which concerns the present work, it has been observed that a normal content in microalgal biomass did not exceed 5% w/w. Indicatively, *Phaeaodactylum tricornutum* yielded 2.2%, *Monodus subterranues* accumulated 2.3 to 3.2% on a dry weight basis, and *Nannochloropsis gaditana* produced biomass with 2.84% of EPA [29,30,31].

**Figure 3:**
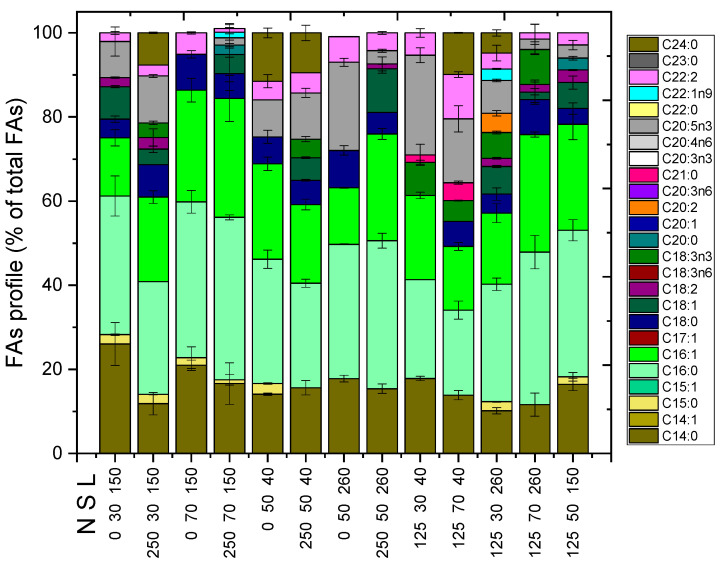
Qualitative profile presentation of the FAs detected, expressed as percentages of the total. N, S, and L on the x-axis stand for nitrogen (mg L^−1^), salinity (ppt), and light (μmol photons m^−2^ s^−1^).

The large variety of FAs (Figure 3) in the biomass of *Nephroselmis* sp. can be divided into saturated and unsaturated molecules. As for the saturated FAs, myristic acid (C14:0), palmitic acid (C16:0), and stearic acid (C18:0) were mainly detected. The first constituted 20% of the total profile, while the second could correspond to 30%, particularly when nitrogen was not supplied to the substrate. Conversely, saturated C18:0 appeared in smaller quantities (<10 %). Looking at the unsaturated FAs, the ones that stood out were palmitoleic acid (C16:1), oleic acid (C18:1), and eicosapentaenoic acid (EPA). One in five FAs was C16:1, while C18:1 appeared at a frequency of about 1 in 10 in every experimental culture. Finally, the EPA was shown to be increased (23.7%) in the case of *Nephroselmis* sp. being provided to a medium with a nitrogen concentration of 125 mg L^−1^ at low salinity levels and under the minimum tested illumination. A large percentage was also occupied when exposed to higher salinity, equal to 70 ppt. In this case, it was measured to be 15.2%. From Figure 3, it was also found that high light intensities (260 μmol m^−2^ s^−1^) did not enhance the accumulation of EPA, which only in the case of zero nitrogen and average salinity approached 11% among the other FAs.

By comparing the FAs profile of the local isolate with that of *Nephroselmis* sp. KGE2, both similarities and differences were distinguished, concluding that the properties of the substrate determined the qualitative and quantitative compositions of the lipid load of the biomass [17]. When *Nephroselmis* sp. KGE2 was cultivated in livestock wastewater, the FAs profile also showed C16:0 in a percentage close to 30%. Contrary to the case of the local isolate, both C14:0 and C18:0 were not found. On the other hand, the cultures from the livestock wastewater effluent exhibited linolelaidic acid (C18:2), which was the most abundant (around 20%) among the other unsaturated FAs, while in the present work, it did not exceed 3% of the total. Finally, the local *Nephroselmis* sp. accumulated α-linolenic acid (C18:3n3) up to 8.3%, whilst *Nephroselmis* sp. KGE2 had a similar content of γ-linolenic acid [17]. 

### 2.2. Other Compounds of Interest

**Figure 4:**
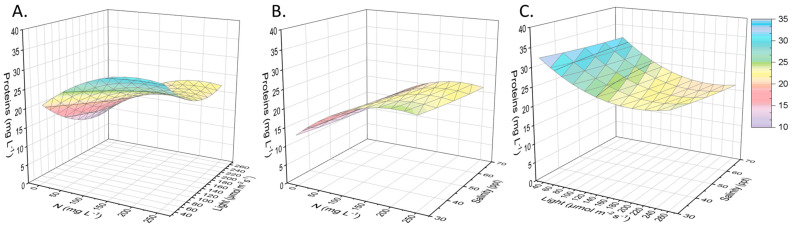
Surface plots regarding proteins as a function of the initial nitrogen concentration, light intensity, and salinity. (**A**) Proteins (mg L^−1^) depending on nitrogen concentration (mg L^−1^) and light intensity (μmol of photons m^−2^ s^−1^), given salinity of 50 ppt. (**B**) Proteins (mg L^−1^) depending on nitrogen concentration (mg L^−1^) and salinity (ppt), given light intensity of 110 μmol of photons m^−2^ s^−1^. (**C**) Proteins (mg L^−1^) depending on salinity (ppt) and light intensity (μmol of photons m^−2^ s^−1^), given nitrogen concentration of 125 mg L^−1^.

The local isolate, Nephroselmis sp., was first grown autotrophically in a complete medium and then inoculated in substrates with different combinations of nitrogen availability, salinity, and light supply. A biomass analysis was carried out in terms of its components for all the examined cases in order to determine the protein, carbohydrate, and pigment contents alongside the lipids. The equations, which describe the various compounds’ content in biomass, are presented in Table 2 and take into account the cases of using all the parameters and only the significant ones. After biomass drying and analysis, it was found that sometimes the carbohydrates or proteins were the main cellular component in direct dependence on the cultivation conditions. Proteins approached 40% of the dry biomass, forming a protein concentration in the culture medium equal to 32 mg L^−1^ when Nephroselmis sp. was exposed to low salinity (30 ppt) and low light radiation (40 μmol m^−2^ s^−1^) in a substate with approximately 200 mg N L^−1^ (Figure 4). Nitrogen is necessary for the production of proteins intracellularly, given that it is also an important component of amino acids. It has been established that the lack of nitrogen affects the photosynthetic activity of microalgae, and the reduction in the growth rate comes together with the low protein content of the biomass. Apart from the available nitrogen, light intensity affects protein synthesis; however, the positive or negative effects of the increase in light radiation depend on the cultivated species. When the light supply was enhanced, Chlorella vulgaris, Desmodesmus sp., and Scenedesmus obliquus produced more proteins, while protein formation by Botryococcus braunii was inhibited [32,33]. Additionally, excessive salinity induces the production of both membrane transport proteins and some plasma proteins in the halotolerant species, such as Chlamydomonas reinhardtii and Dunaliella salina [34].

**Figure 5:**
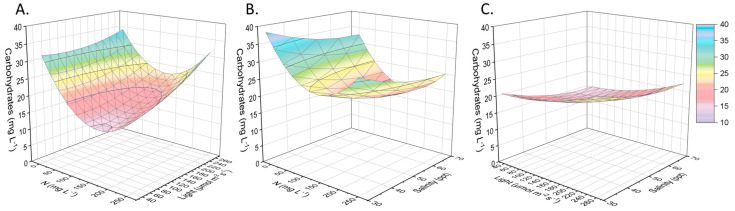
Presentation of the surface plots regarding carbohydrate production as a function of the initial nitrogen concentration, light intensity, and salinity. Carbohydrates (mg L^−1^) related to (**A**) nitrogen concentration (mg L^−1^) and light intensity (μmol of photons m^−2^ s^−1^), given salinity of 50 ppt. (**B**) Nitrogen concentration (mg L^−1^) and salinity (ppt), given light intensity of 110 μmol of photons m^−2^ s^−1^. (**C**) Salinity (ppt) and light intensity (μmol of photons m^−2^ s^−1^), given nitrogen concentration of 125 mg L^−1^.

**Figure 6:**
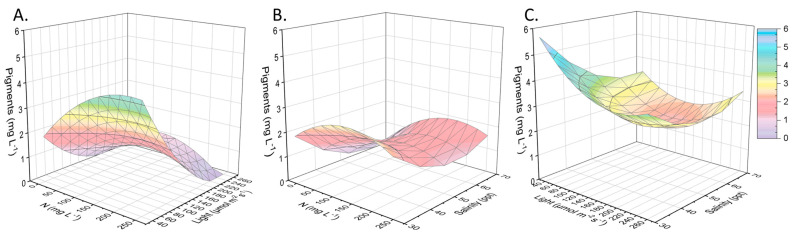
Presentation of the surface plots regarding pigments production (chlorophyll a, chlorophyll b and total carotenoids) production as a function of the initial nitrogen concentration, light intensity, and salinity. Pigments (mg L^−1^) related to (**A**) nitrogen concentration (mg L^−1^) and light intensity (μmol of photons m^−2^ s^−1^), given salinity of 50 ppt. (**B**) Nitrogen concentration (mg L^−1^) and salinity (ppt), given light intensity of 110 μmol of photons m^−2^ s^−1^. (**C**) Salinity (ppt) and light intensity (μmol of photons m^−2^ s^−1^), given nitrogen concentration of 125 mg L^−1^.

In contrast, intracellular carbohydrates were favored by nitrogen stress and high light intensity. Aside from nutrient starvation, the international literature confirms high salinity and excessive illuminance as methods for the accumulation of intracellular sugars [35]. More specifically, the sugars reached 45% (40 mg L*^−^*^1^), being the predominant compound (Figure 5). Nephroselmis sp., as eukaryotic microalgae, produces chlorophyll b in addition to chlorophyll a and carotenoids, and no genes for phycobilins are expressed. As shown in Figure 6, when the local isolate was adapted to an environment with sufficient nitrogen (around 200 mg L*^−^*^1^) and little light, the total pigments increased intracellularly to 7% *w/w* (6 mg L*^−^*^1^). When the light was in excess, photosynthetic pigments (chlorophyll and carotenoids) normally decreased, while the secondary carotenoids increased in some chlorophytes for photoprotection purposes [36]. Species belonging to Chlorophyta produced lutein and zeaxanthin, apart from b-carotene. Nephroselmis sp., in particular, was characterized by a high content of zeaxanthin that can represent more than 50% of the total carotenoids [37]. 

## 3. Materials and Methods

### 3.1. Nephroselmis sp. Cultivation Conditions 

The local isolate *Nephroselmis* sp., obtained from the saltworks (Messolonghi, Western Greece) [6], grew autotrophically, and a two-stage cultivation mode followed. Τhe experimental conditions are based on preliminary works [6,8]. The first cultivation was carried out in a 6 L conical flask. Walne’s medium was used to produce a total of 1.2 g biomass (200 mg L^−1^) from an initial *Nephroselmis* sp. inoculum of 300 mg (50 mg L^−1^). Cell proliferation was autotrophic under the illumination of 100 μmol of photons m^−2^ s^−1,^ and the aeration was regulated at a flow rate of 1.5 L_air_ min^−1^ L_culture_ so that the medium was saturated in carbon dioxide to avoid oxygen accumulation. 

### 3.2. Second Stage Cultivation: Experimental Design and Conditions

Regarding the added-value compounds accumulation in the second stage, a three-level Box–Behnken experimental design was applied to three variables (nitrogen, salinity, and light intensity) using Minitab 19. The aforementioned variables were adjusted to 0–250 mg N L^−1^, a salinity of 30–70 ppt, and light intensity of 40–160 μmol m^−2^ s^−1^ in 15 tests (in duplicate) of 400 mL each, as described in detail in Table 1. For the presented diagrams and behavior prediction of each compound (or compounds group) of interest, two types of equations are provided in Table 2. Such equations refer to the case of taking into consideration all the parameters tested (and their combinations), as well as the case of using only the parameters of statistical significance (simplified form), as derived from the Pareto charts, which are presented in Supplementary Materials.

### 3.3. Analytical Methods

#### 3.3.1. Determination of Harvested Biomass, Total Solids, Volatile Solids, and Moisture

The harvested microalgal biomass was primarily washed, freeze-dried (Telstar, LyoQuest, Madrid, Spain), and estimated as a dry cell weight per volume of culture, according to the standard methods [38] for total suspended solids (TSS) determination, while the measurement of the compounds of interest followed. The calculation of total solids (TS), volatile solids (VS), and moisture was accomplished according to the standard methods [38].

#### 3.3.2. Fatty Acids Determination 

The FAs measurement was conducted on samples of 10–40 mg dry biomass, after their in-situ transesterification reaction, in the presence of the H_2_SO_4_ catalyst and the use of 5 mL of 100:10:1 CH_3_OH:CHCl_3_:H_2_SO_4_ solution. The samples were incubated for 2 h at 90 °C, and the addition of 2 mL H_2_O and 2 mL of 4:1 C_6_H_14_:CHCl_3_ followed for the stop of the transesterification reaction and the FAMEs extraction, respectively. The analysis was performed using gas chromatography with a flame ionization detector and He as the carrier gas (Agilent Technologies, 7890A, Wilmington, DE ,USA); however, more information regarding the process can be found in Koutra et al. [39]. 

#### 3.3.3. Determination of Pigments, Proteins, and Carbohydrates Production

Additionally, the extraction of dry biomass by N, N′-dimethylformamide was accomplished for the pigments determination (chlorophyll a, b, and total carotenoids). The calculations for the pigments can be found in the work of Mastropetros et al. [40]. The protein content was quantified by the semi-micro Kjeldahl method [38], and their conversion to proteins was followed by multiplying the total Kjeldahl nitrogen by a factor of 6.25. Finally, the carbohydrates were determined colorimetrically through the Dubois method [41].

### 3.4. Analysis of Variance

The collected biomass from the second stage of cultivation was statistically analyzed through analysis of variance (ANOVA), while t-tests were conducted for comparison purposes. The statistical analysis and the data grouping according to their statistical difference were carried out via the Minitab 19 software (Minitab LLC, State College, PA, USA).

## 4. Conclusions

The results of the present study strengthened the position of microalgae in the field of nutritional biomass production and, more specifically, in the recovery of valuable FAs. The microalgae of the *Nephroselmis* genus are not widely examined regarding the accumulation of FAs and other biomass compounds, such as proteins, carbohydrates, and pigments, compared to other more common species. The local isolate, *Nephroselmis* sp., produced up to 15 mg L^−1^ of FAs, of which up to 30% may comprise polyunsaturated EPA. Regardless of the cultivation conditions, the FAs of the local microalgae were mainly C14:0, C16:0, C16:1, and C20:5n3 (EPA). Their amount increased in the presence of nitrogen (210 to 220 mg L^−1^), at low salinity levels (30 ppt), and an illumination ranging from 150 to 170 μmol m^−2^ s^−1^. Nitrogen deficiency did not favor the accumulation of FAs, as an adaptation of the cells to the new batches required lipids consumption for their energy purposes. Finally, *Nephroselmis* sp. was characterized by a high protein content when the nitrogen was adequate; however, proteins were replaced by intracellular sugars in the absence of nitrogen. The pigments from this species were maximized after five days in media with low salinity under a light intensity that was limited below 60 μmol m^−2^ s^−1^.

## Figures and Tables

**Table 1:** Acquired data after the Box–Behnken experimental design and presentation of the biomass concentration after 5 days of cell acclimation. The standard deviations arose from the differences between the two replicate cultures, and values that share a letter are not significantly different (*p* = 0.05).

Run	Nitrogen (mg L^−1^)	Salinity (ppt)	Light (μmol m^−2^ s^−1^)	Biomass (mg/L) (Average ± SD)
1	0	30	150	92.9 ± 22.5 ^A^
2	250	30	150	117.4 ± 8.1 ^A^
3	0	70	150	96.3 ± 8.6 ^A^
4	250	70	150	109.8 ± 5.6 ^A^
5	0	50	40	103.2 ± 1.4 ^A^
6	250	50	40	97.2 ± 9.4 ^A^
7	0	50	260	106.2 ± 11.4 ^A^
8	250	50	260	113.2 ± 9.8 ^A^
9	125	30	40	98.4 ± 0.9 ^A^
10	125	70	40	96.4 ± 6.1 ^A^
11	125	30	260	111.1 ± 0.3 ^A^
12	125	70	260	127.1 ± 26.5 ^A^
13	125	50	150	108.0 ± 20.9 ^A^
14	125	50	150	114.4 ± 1.4 ^A^
15	125	50	150	101.7 ± 2.2 ^A^

**Table 2:** Equation presentation for the description of biomass compounds production in the tested range of nitrogen (0–250 mg L^−1^), light intensity (40–260 μmol of photons m^−2^ s^−1^), and salinity (30–70 ppt). The equations are provided in their analytical form, considering all parameters, as well as in a simplified form, taking into account only the parameters of statistical significance, as derived by Minitab 19.

Biomass Compounds	Equation (All Parameters)	Simplified Equation (Only Parameters of Statistical Significance)
Proteins (mg/L)	$\begin{array}{c} 28.7+1.05{\times 10}^{-1}N-0.88{\times 10}^{-1}S\\ \;\;\;\;\;\;-1.32{\times 10}^{-1}L-2.58{\times 10}^{-4}N^{2}\\ \;\;\;\;\;\;+0.66{\times 10}^{-3}S^{2}+3.39{\times 10}^{-4}L^{2}\\ \;\;\;\;\;\;-0.51{\times 10}^{-4}NS+0.14{\times 10}^{-4}NL\\ \;\;\;\;\;\;-2.17{\times 10}^{-4}SL \end{array}$	$\begin{array}{c} 28.7+1.05{\times 10}^{-1}N-1.32{\times 10}^{-1}L\\ \;\;\;\;\;\;-2.58{\times 10}^{-4}N^{2}\\ \;\;\;\;\;\;+3.39{\times 10}^{-4}L^{2} \end{array}$
Carbohydrates (mg/L)	$\begin{array}{c} 52.5-2.17{\times 10}^{-1}N-6.15{\times 10}^{-1}S\\ \;\;\;\;\;\;-2.05{\times 10}^{-2}L+6.52{\times 10}^{-4}N^{2}\\ \;\;\;\;\;\;+4.39{\times 10}^{-3}S^{2}+1.38{\times 10}^{-4}L^{2}\\ \;\;\;\;\;\;-0.48{\times 10}^{-4}NS\\ \;\;\;\;\;\;+1.71{\times 10}^{-4}NL\\ \;\;\;\;\;\;+1.65{\times 10}^{-4}SL \end{array}$	$\begin{array}{c} 52.5-2.17{\times 10}^{-1}N-6.15{\times 10}^{-1}S\\ \;\;\;\;\;\;-2.05{\times 10}^{-2}L\\ \;\;\;\;\;\;+6.52{\times 10}^{-4}N^{2} \end{array}$
Pigments (mg/L)	$\begin{array}{c} 11.32+2.07{\times 10}^{-2}N-2.69{\times 10}^{-1}S\\ \;\;\;\;\;\;-3.93{\times 10}^{-2}L-0.48{\times 10}^{-4}N^{2}\\ \;\;\;\;\;\;+2.01{\times 10}^{-3}S^{2}+0.08{\times 10}^{-4}L^{2}\\ \;\;\;\;\;\;+0.64{\times 10}^{-4}NS\\ \;\;\;\;\;\;-0.61{\times 10}^{-4}NL\\ \;\;\;\;\;\;+2.19{\times 10}^{-4}SL \end{array}$	$\begin{array}{c} 11.32+2.07{\times 10}^{-2}N-2.69{\times 10}^{-1}S\\ \;\;\;\;\;\;-3.93{\times 10}^{-2}L\\ \;\;\;\;\;\;-0.48{\times 10}^{-4}N^{2}\\ \;\;\;\;\;\;+2.01{\times 10}^{-3}S^{2}\\ \;\;\;\;\;\;+0.08{\times 10}^{-4}L^{2}\\ \;\;\;\;\;\;-0.61{\times 10}^{-4}NL\\ \;\;\;\;\;\;+2.19{\times 10}^{-4}SL \end{array}$
FAs (mg/L)	$\begin{array}{c} 7.20+6.86{\times 10}^{-2}N-1.03{\times 10}^{-1}S\\ \;\;\;\;\;\;+6.81{\times 10}^{-2}L-1.28{\times 10}^{-4}N^{2}\\ \;\;\;\;\;\;+1.79{\times 10}^{-3}S^{2}-0.17{\times 10}^{-3}L^{2}\\ \;\;\;\;\;\;-5.23{\times 10}^{-4}NS\\ \;\;\;\;\;\;+0.13{\times 10}^{-4}NL\\ \;\;\;\;\;\;-4.86{\times 10}^{-4}SL \end{array}$	$7.20+6.86{\times 10}^{-2}N$
EPA (% of FAs)	$\begin{array}{c} 51.3+4.47{\times 10}^{-2}N-1.15S-1.87{\times 10}^{-1}L\\ \;\;\;\;\;\;-0.93{\times 10}^{-4}N^{2}\\ \;\;\;\;\;\;+9.09{\times 10}^{-3}S^{2}+4.53{\times 10}^{-4}L^{2}\\ \;\;\;\;\;\;-0.83{\times 10}^{-3}NS\\ \;\;\;\;\;\;-0.92{\times 10}^{-4}NL\\ \;\;\;\;\;\;+3.62{\times 10}^{-4}SL \end{array}$	$\begin{array}{c} 51.3-1.15S-1.87{\times 10}^{-1}L+9.09{\times 10}^{-3}S^{2}\\ \;\;\;\;\;\;+4.53{\times 10}^{-4}L^{2} \end{array}$

*N*: nitrogen (mg L^−1^), *L*: light intensity (μmol of photons m^−2^ s^−1^), and *S*: salinity (ppt).

## Data Availability

Available on request.

## Supplementary Materials

The following supporting information can be downloaded at: https://www.mdpi.com/article/10.3390/md21060331/s1, Figure S1: Pareto charts for the statistical significance of the nitrogen concentration, salinity level, and illuminance regarding the derived equations for (a) proteins, (b) carbohydrates, (c) pigments, (d) FAs, and (e) EPA production.
