# Supplementary material for: Effect of Nitrogen, Salinity, and Light Intensity on the Biomass Composition of Nephroselmis sp.: Optimization of Lipids Accumulation (Including EPA)
Source: Mar Drugs. 2023 May 28;21(6):331. doi: 10.3390/md21060331 (PMC10301981; doi:10.3390/md21060331)
Supplement: Supplementary file 1 [file marinedrugs-21-00331-s001.zip › marinedrugs-2419903-supplementary.pdf]

Supplementary materials

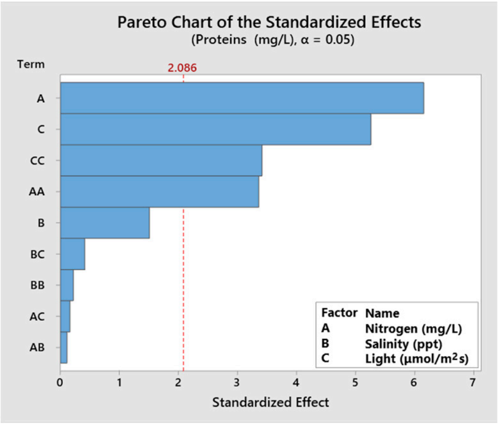

(a)

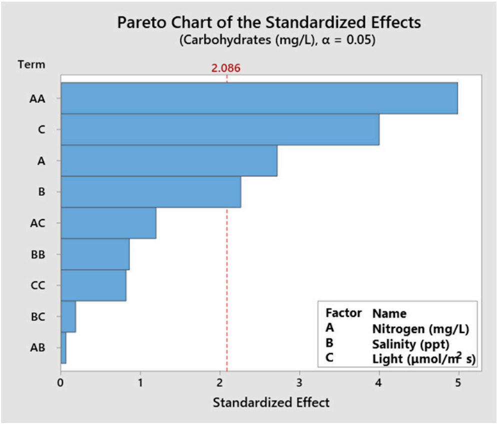

(b)

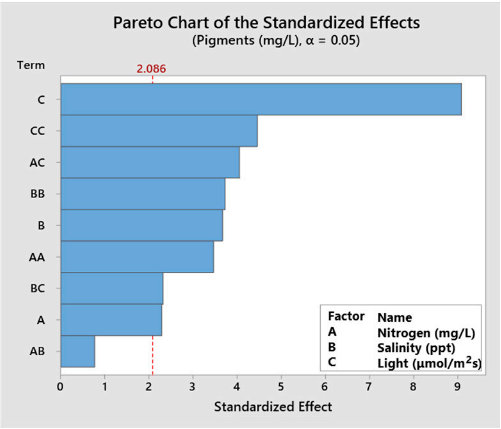

(c)

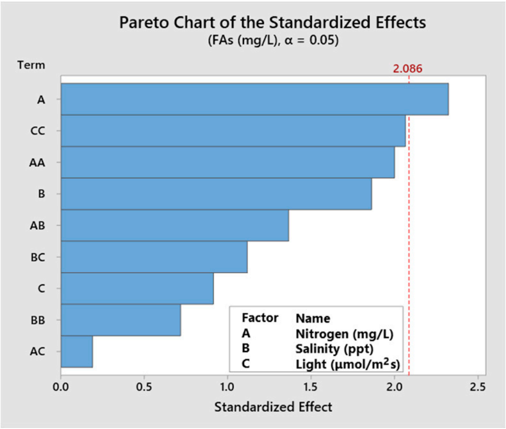

(d)

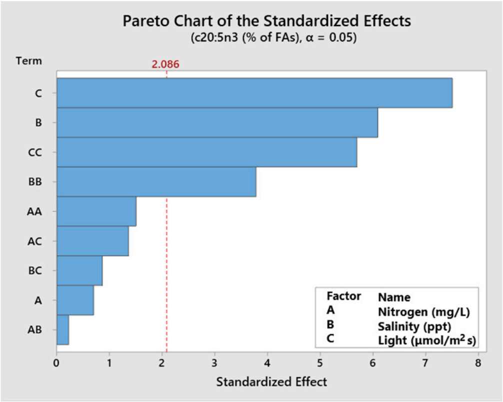

(e)

Figure S1. Pareto charts for the statistical significance of the nitrogen concentration, salinity level and illuminance, regarding the derived equations for (a) proteins, (b) carbohydrates, (c) pigments, (d)fatty acids and (e) EPA production.
